# Voltage-controlled NiO/ZnO p–n heterojunction diode: a new approach towards selective VOC sensing

**DOI:** 10.1038/s41378-020-0139-1

**Published:** 2020-06-01

**Authors:** Sayan Dey, Swati Nag, Sumita Santra, Samit Kumar Ray, Prasanta Kumar Guha

**Affiliations:** 1 Department of Electronics & Electrical Communication Engineering, Indian Institute of Technology, Kharagpur, 721302 India; 2 Department of Electrical Engineering, National Institute of Technology, Agartala, 99046 India; 3 Department of Physics, Indian Institute of Technology, Kharagpur, 721302 India; 4 Department of Physics, Belda College, Belda, 721424 India; 5 S N Bose National Centre for Basic Sciences, Kolkata, 700106 India

**Keywords:** Electrical and electronic engineering, Environmental, health and safety issues

## Abstract

Metal oxide resistive gas sensors suffer from poor selectivity that restricts their practical applicability. Conventional sensor arrays are used to improve selectivity which increased the system complexity. Here, we have proposed a novel NiO/ZnO-based p–n junction single-diode device for selective sensing of several volatile organic compounds (VOCs) simultaneously by tuning bias voltage. The operating voltage was varied between 3 and 5 volts to achieve selective sensing of 2-propanol (19.1 times for 95 ppm with response and recovery times of 70 s and 55 s respectively‚ at 3 volts), toluene (20.1 times for 95 ppm with response and recovery times of 100 s and 60 s respectively, at 4 volts), and formaldehyde (11.2 times for 95 ppm with response and recovery times of 88 s and 54 s respectively, at 5 volts). A probable mechanism of the tunable selectivity with operating bias voltage due to increase in surface carriers with increasing voltage was hence put forth. Thus, this device may play an important role to develop future selective multiple VOC sensor thereby replacing standard sensor arrays.

## Micro gas sensors: novel diodes for simultaneous detection of multiple volatile organic compounds

Employed in manufacturing many industrial products, volatile organic compounds (VOCs) are often highly toxic. A diode based device has been developed that allows the selective sensing of several VOCs simultaneously in a cost-efficient manner. Various methods can be used to detect VOCs, but they have considerable shortcomings, including high cost, excessive size, and poor selectivity. However, a team headed by Prasanta Kumar Guha at the Indian Institute of Technology Kharagpur, India has succeeded in developing a new single-diode device based on nickel oxide and zinc oxide (NiO/ZnO) heterojunction that can be fabricated in an environment-friendly fashion and allows accurate sensing of VOCs. The authors believe that their voltage-tunable selective NiO/ZnO diode device has considerable potential for application as future VOC sensors, replacing the complex gas sensor arrays that are currently in standard commercial use.

## Introduction

Volatile organic compounds (VOCs) are used for the development of several industrial products^1^. However, VOCs are toxic and can cause many short-term as well as long-term health problems if inhaled in excess amount^2–5^. There are several applications where selective detection of VOCs is important. For example, detection and removal of VOCs (source: consumer products—formaldehyde, benzene, toluene, ethyl benzene, and xylene) are necessary in smart houses to maintain indoor air quality^6^. Specific VOCs (in excess amount) can be found in exhaled human breath during deteriorated health condition, e.g., acetone for type II diabetes mellitus, ammonia for renal failure, and NO_x_ for asthma and COPD. Detection of VOCs plays an important role in agriculture as well, e.g., ethylene and esters are released with ripening of fruits (e.g., banana). So, level of such VOCs can give information of ripening stage of fruits. Thus, it is important to detect VOCs selectively^7,8^.

VOCs can be detected using various techniques. For example, gas chromatography–mass spectrometry (GC–MS) is an important tool to detect VOCs very accurately. But, it is bulky, trained man power is required, data cannot be obtained in real time, and also GC–MS is hugely expensive (>$50,000). Other sensors, like optical, electrochemical, and resistive sensors are also being used for detecting VOCs. Optical sensors (e.g., infrared), though accurate, are very expensive (>$1000). Electrochemical sensors are also not cheap (each discrete sensor may cost ~$25), have larger footprint and also suffer from short life time (typically 1 year). Resistive sensors have much smaller footprint due to matured microelectronics technology but they suffer from poor selectivity. There are primarily two ways through which sensors’ selectivity can be improved—(i) using functionalized nanomaterial-based sensing layer and (ii) using pattern recognition techniques. In the first approach, metal oxides are doped with metal nanoparticles (e.g., SnO_2_ or ZnO or WO_3_ with Pt, Pd, and Au nanoparticles)^9–11^, or composites are formed with other metal oxides or two-dimensional materials (e.g., SnO_2_, ZnO with graphene or MoS_2_)^12–16^. This approach though improves the sensor performance but fails to make sensors fully selective. On the other hand, pattern recognition algorithm can also be used to train sensors and then (using database) detect the unknown VOCs^17^. This is more effective for improving selectivity; however, the approach is complicated and sometimes computer intensive. In fact, most of the VOC sensors available in the market today gives information of total VOC, instead of individual VOC. In this respect, p–n heterojunction diode can play an important role. A p–n diode can also be integrated with CMOS (complementary metal oxide semiconductor) die-like resistive sensor. But the main advantage is, a diode forward bias voltage can be changed to modulate the number of carriers participating in sensing. We believe this can be used to tune the selectivity of the sensor among different VOCs. A p–n nano heterojunction can be formed (both in situ and ex situ) in the crystallite level as well. Several reports demonstrate p–n nano heterojunctions (e.g., NiO-ZnO^18,19^ and NiO-graphene^20^) as sensors. However, the diode like nonlinear nature is not expected in these cases as it is impossible to take contacts individually from p and n sides and hence the voltage-tunable selectivity cannot be achieved. These films hence behave as conventional resistive sensors. To the best of authors’ knowledge, this will be the first report of voltage control selectivity.

In this work, NiO/ZnO-based hetero p–n junction diode was fabricated via environment friendly chemical route for selective detection of VOCs. Here, NiO was fabricated by liquid exfoliation, followed by growth of ZnO nano-forest using low temperature hydrothermal technique. The heterojunction device showed excellent response toward three VOCs. In fact, the sensor showed specific sensing toward 2-propanol, toluene, and formaldehyde at operating voltage of 3, 4, and 5 volts, respectively. The operating temperature was found to be optimum at 300 °C for all the three VOCs. A T-CAD simulation of sensor device was carried out to support the experimental data. A probable sensing mechanism is proposed for the voltage-tunable selectivity of the sensor.

## Materials and methods

### Fabrication of NiO/ZnO heterojunction device

The sensor device was fabricated by evenly growing ZnO hierarchical nanostructures on NiO nano flakes. All chemicals were purchased from Merck India Pvt. Ltd. and were used without further purification. In a typical fabrication process, 0.05 M Ni(NO_3_)_2_·6H_2_O was solubilized in 10 mL deionized (DI) water. To this, 10 mL of 0.1 M NaOH was added dropwise under constant stirring. The resultant precipitate was washed and filtered to obtain pure Ni(OH)_2_ that was then dispersed in 20 mL dimethylformamide and sonicated for 8 h to obtain single to few layered flakes of Ni(OH)_2_. Then Ni(OH)_2_ film was spin coated on a ITO coated glass.

**Fig. 1:**
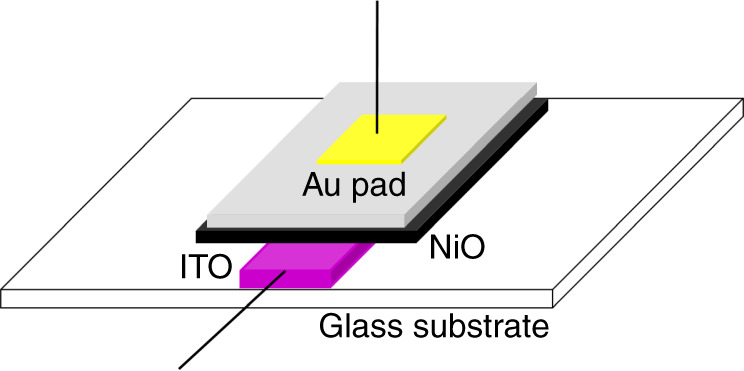
Schematic of NiO/ZnO heterojunction-based gas sensor device; a vertical device with two electrodes, i.e., top (Au) and bottom (ITO). NiO (p-type) layer is beneath ZnO (n-type) layer. ZnO surface is defect induced to enhance gas sensing

The ZnO nanostructure layer was grown hydrothermally on Ni(OH)_2_ film. For growing ZnO, 0.05 M Zn(NO_3_)_2_·6H_2_O were dissolved in 120 mL DI water. To this, 6 mL 25% NH_3_ solution was added under constant stirring until the turbidity disappears. The solution was then transferred to a teflon-lined steel autoclave and the Ni(OH)_2_ coated substrate was obliquely immersed in the solution. The solution was then kept at 95 °C for 3 h. The autoclave was allowed to cool at room temperature, and the substrate was washed and dried. Finally, the device was calcined at 450 °C and gold contacts were thermally evaporated to form NiO/ZnO heterojunction-based sensing device. The schematic of the device is illustrated in Fig. 1.

### Gas sensing measurement

The sensing experiment was carried out in a customized air tight gas test setup. The sensor was interfaced with a semiconductor parametric analyzer (Agilent 4156 C along with a test fixture) to measure the real-time change in sensor signal (current). The flow of VOC and dry air were controlled by digital mass flow controllers (MFC: Alicat Inc.). A detailed description of the setup was demonstrated in ref. ^21^. The baseline of sensor was stabilized by purging dry air for 2 h and then different concentrations (19–95 ppm) of VOCs (viz. 2-propanol, toluene, and formaldehyde) were introduced into the chamber. The operating diode voltage was varied from 3 to 5 volts. The response was calculated by the following formula:

1 $${\mathrm{Response}} = \left( {\frac{{I_{{\mathrm{VOC}}}}}{{I_{\mathrm{a}}}}} \right)$$

where, *I*_a_ is the current through the sensor in presence of dry air and *I*_VOC_ is the current through the sensor after introduction of VOCs diluted by air.

## Results and discussions

### Sensing device characterization

**Fig. 2:**
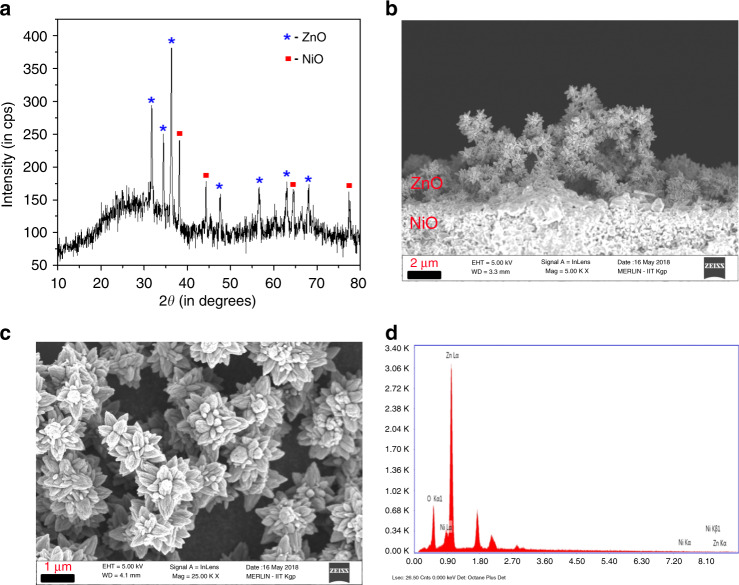
Crystallographic and morphological characterization of the sensor device. **a** XRD spectrum of NiO/ZnO heterojunction showing distinct peaks of NiO (marked with red ligands) and ZnO (marked with blue ligands), supporting coexistence of the two oxides. Low intense peaks of NiO depict the relative position of NiO w.r.t. ZnO surface **b** FESEM image (sectional view) showing the heterojunction formation. Clear junction between NiO (flakes) and ZnO (hierarchical nanostructures) on top can be observed **c** FESEM image (top view) showing formation of ZnO hierarchical nanostructures on NiO flakes. The dark areas depict NiO **d** EDAX spectrum of the NiO/ZnO heterojunction showing top layer ZnO (intense peak of Zn) and bottom layer NiO (comparatively less intense peak of Ni)

The phase and crystalinity of the device were performed by X-ray diffraction (XRD; Ultima III, Rigaku X-ray diffractometer; Cu *K*_α_ radiation, *λ* = 1.5404 Å). The XRD spectrum shows peaks at 38.12°, 44.35°, 64.62°, and 77.45° corresponding to (111), (200), (220), and (311) of NiO (JCPDS card no. 47-1049) and 31.88°, 34.59°, 36.31°, 47.70°, 56.56°, 62.99°, and 68.13° corresponding to (100), (002), (101), (102), (110), (103), and (112) of hexagonal ZnO (JCPDS card no. 36-1451; shown in Fig. 2a). Absence of any other peaks confirms the formation of pure crystalline phases of NiO and ZnO only.

The morphological study of the prepared device was performed by FESEM imaging (shown in Fig. 2b, c) and elemental composition analysis was done by EDAX (Fig. 2d). Figure 2b shows the formation of the p–n NiO/ZnO heterojunction, while Fig. 2c shows the flower like morphology of ZnO on flake like NiO nanostructures. The average thickness of both NiO and ZnO layers are ~2 µm. The EDAX analysis shows the existence of Ni, Zn, and O in the nanostructure thereby confirming the elemental composition of the so-formed heterojunction.

### Gas sensing results

**Fig. 3:**
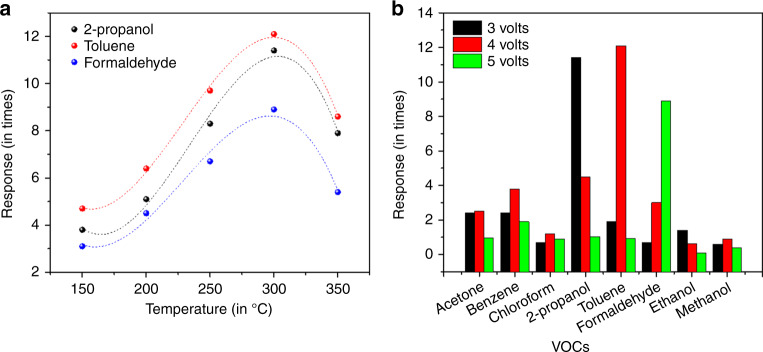
Sensor operating parameter optimization. **a** Temperature profiles for three different VOCs. Only the VOCs with maximum response was chosen. A clear maxima at 300°C can be observed for all the three VOCs. **b** Specificity of sensor device for different VOCs with varying operating voltage (3–5 volts). Clear maximas were observed for three different VOCs at three different forward bias voltage

The sensor device was exposed to 2-propanol, toluene, and formaldehyde vapors (19 ppm to 95 ppm), respectively. The maximum response was obtained at 300 °C for all three VOCs, hence all the experiments were performed at 300 °C (Fig. 3a). The diode was biased at three different voltages (3, 4, and 5 volts). The sensor showed maximum response toward 2-propanol at 3 V, toluene at 4 V, and formaldehyde at 5 V (Fig. 3b). The sensor was exposed to other VOCs also, but showed specificity of response only to the above three VOCs mentioned at those voltages.

**Fig. 4:**
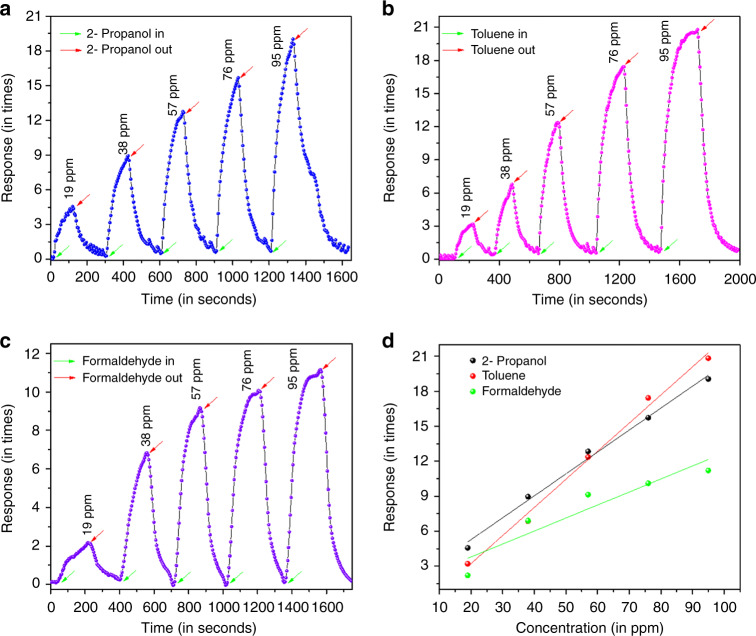
Gas sensing performance of NiO/ZnO heterojunction based device for different VOCs at different bias voltage. The plots for **a** 2-propanol at 3 volts, **b** toluene at 4 volts, and **c** formaldehyde at 5 volts show complete recovery for each VOC. **d** Response vs. concentration plot of the three VOCs

The gas sensor showed excellent response toward three VOCs. The sensor was found to give 19.1 times response with 95 ppm of 2-propanol with response and recovery times of 70 s and 55 s respectively, at an operating voltage of 3 volts (Fig. 4a). When the operating voltage was increased to 4 volts, the device sensed 95 ppm toluene (response 20.1 times) with response and recovery times of 100 s and 60 s respectively (Fig. 4b), while the specificity changed to formaldehyde (response 11.2 times) with an operating voltage of 5 volts and response and recovery times of 88 s and 54 s respectively (Fig. 4c). The Fig. 4d demonstrated the linear nature of the sensor response with respect to change in concentration of VOC vapors.

The limit of detection (LOD) of the sensor for the different VOCs was calculated using the following relation^22,23^:

2 $${\mathrm{LOD}} = \left( {\frac{{3\sigma _{{\mathrm{noise}}}}}{s}} \right)$$

where, *σ*_noise_ is the standard deviation (root mean square) of noise and *s* is the slope of the response vs. concentration plot. The calculated value of LOD for the sensor was 710 ppb, 1.92 ppm, and 961 ppb for 2-propanol, toluene, and formaldehyde, respectively.

It was observed that the sensor was selective for 2-propanol over similar alcohols (methanol, ethanol, etc.), which should undergo same oxidation reaction under similar physical conditions. This could be explained on the basis of the electron withdrawing nature (−I effect) of the functional groups. In general, the four functional groups encountered in the present study can be arranged as follows:

–CHO > –OH > –C_6_H_5_ > –R. Hence, it can be concluded that the the oxygen species requirement by the above groups for any reaction (in this case oxidation) will be more for –CHO (aldehyde group, in this case HCHO) and least for –R (alkyl group, in this case methanol and ethanol). Again, considering electron releasing nature (+I effect) of the alkyl groups, the arrangement would be: 3° > 2° > 1°. 2-Propanol being a 2° alcohol, while methanol and ethanol are 1° alcohols, the oxygen requirements for these structurally different alcohols are not same. 2-Propanol, having a higher +I effect will release less electrons during reaction.

**Fig. 5:**
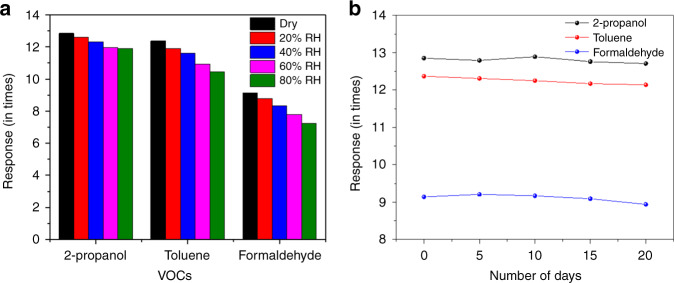
Device stability study for the heterojunction device under real-time environment. Plots showing the **a** effect of humidity (varied from 20 to 80% RH) on the performance of the NiO/ZnO sensor device. **b** Device stability analysis over a period of 3 weeks. All measurements were performed at a VOC concentration of ~57 ppm

The sensor performance was studied in varying humidity (20–80% RH). It was observed that there was a slight degradation in the sensor response (11.91 times for 2-propanol, 10.46 times for toluene, and 7.23 times for formaldehyde; concentration of all VOCs ~ 57 ppm) with respect to dry VOC vapors (Fig. 5a). The sensor was also stable toward varying ambient temperature (varied from 25–125 °C). As the sensor operating temperature was above 200 °C, the effect of ambient temperature could not be identified separately. The sensor was tested for stability as its sensitivity depended on the stability of the thin junction layer. It was found to be stable for 3 weeks (Fig. 5b) without any significant deviation in response.

### Sensing mechanism

**Fig. 6:**
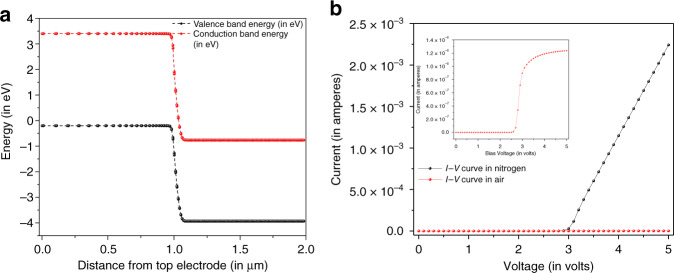
T-CAD simulation results demonstrating device performance. Illustrations show **a** band diagram of NiO/ZnO heterojunction sensor device showing broken type (type III) heterojunction formation. Absence of potential well reduced trapped states thereby increasing the effectiveness of the sensor. **b** Experimental plot of diode current vs. voltage in dry nitrogen (black line) and in dry air (red line), inset is zoomed in view of diode current in dry air
(iv) In order to support the above experimental argument, simulation was carried out of the diode structure by a T-CAD simulation tool (Silvaco). In Fig. 7a, the simulated diode current was plotted against varying surface carrier concentration of ZnO layer for the three supply voltages (3 V, 4 V, and 5 V). It shows that diode current decreases drastically with decrease in surface carrier concentration. Also, the current is higher for larger supply voltage for the same surface carrier concentration. In Fig. 7b, we have plotted the diode current at three voltages in both dry nitrogen and dry air, just to give emphasis in our argument that diode current is greatly suppressed in dry air because of the adsorption of free carriers by oxygen molecules present in dry air. So, it can be concluded that, higher the forward voltage (in dry air) higher will be the O^−^ adsorbates present at the ZnO surface. These O^−^ will interact with the VOCs and release the trapped electrons (see the equations below in (v)), which in turn will increase the diode currents.

The mechanism of tuning selectivity of VOC sensor device by varying bias voltage may be explained as follows:

(i) The band diagram of NiO/ZnO heterojunction device (*E*_gNiO_ = 3.67 eV, electron affinity NiO 3.95 eV and *E*_gZnO_ = 3.24 eV, electron affinity ZnO 4.35 eV) has been illustrated in Fig. 6. From the figure, it was observed that the junction was a broken type (type III) heterojunction where carrier transfer is expected to be more pronounced (compared to if there is a notch present at the junction).
(ii) It is well known that height of a p–n junction barrier potential can be modulated by applying bias voltage. The barrier height reduces on increasing the forward bias, thereby facilitating more carriers (electrons and holes) to cross the junction. The more the number of carriers crossing the junction, the more will be the carriers need to replenish from the bias voltage to maintain the supply of carriers.
(iii) The oxygen molecules (present in air) can absorb the conduction band electrons present near the surface. In this case, free electrons near the top surface of ZnO will be absorbed by oxygen forming O^−^. So, if we increase forward bias voltage, there will be more electrons (from the battery −ve terminal) supplied to the ZnO surface, which in turn gives rise to more number of O^−^ adsorbates. This can be seen from the experimental plot in Fig. 6b. The *I*–*V* characteristics shows much higher diode current in presence of dry N_2_ (black plot) compared to that in dry air (red plot).

**Fig. 7:**
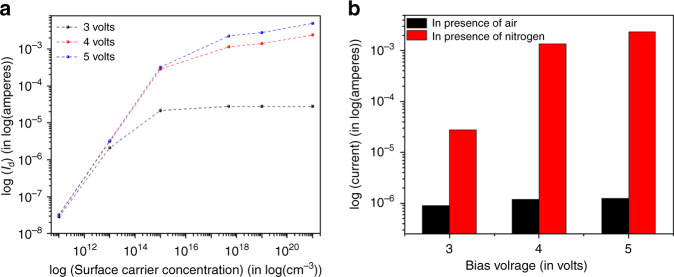
Device performance analysis in support of VOC sensing mechanism. The plots represent **a** T-CAD simulation of diode structure, current vs. surface carrier concentration for three different voltages. A definite variation of surface carrier concentration with increasing forward bias voltage was observed supporting the proposed sensing mechanism. **b** Experimental diode current at three voltages in dry nitrogen and dry air
(v) The equations governing the sensing of the three VOCs are as follows:
  with 2-propanol: (CH_3_)_2_ – CH–OH + O^−^ → (CH_3_)_2_ – CO + H_2_O + 1e^−^
  with toluene: C_7_H_8_ + 2O^−^ → C_7_H_6_O + 2e^−^
  with formaldehyde: HCHO + O^−^ → HCOOH
  $$2{\mathrm{HCOOH}} + 2{\mathrm{O}}^ - \to {\mathrm{CO}}_2 + {\mathrm{H}}_2{\mathrm{O}} + 3{\mathrm{e}}^ -$$
  Thus, from the above equations, it is clear that the requirement of oxygen adsorbates (i.e., availability of electron(s) to form O^−^) increases from 2-propanol to toluene and then highest in case of formaldehyde. This in turn shifts the selectivity from 2-propanol to formaldehyde with increase in operating voltage due to the increase in number of available electrons to take part in the sensing. As mentioned before, on introducing the analyte gas (having different oxygen radical requirement), the adsorbed oxygen species get desorbed upon reaction with the analyte thereby repopulating the surface with electrons. As a result, the diode current increases. This is illustrated in Fig. 8a.

**Fig. 8:**
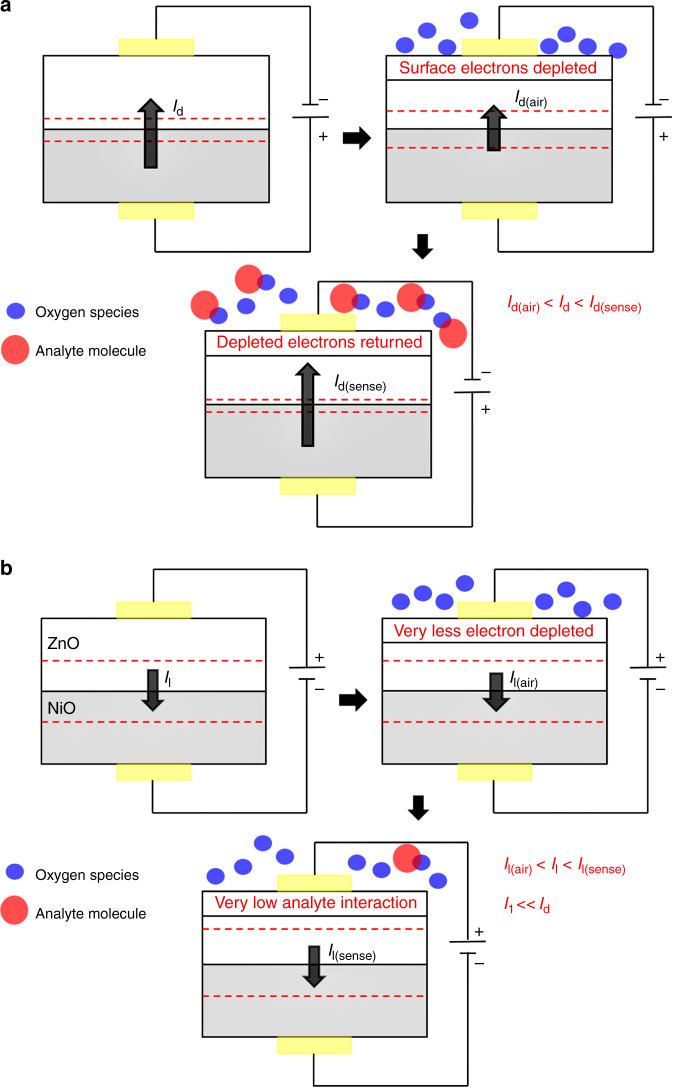
Movement of current in the p–n junction device in absence and presence of oxygen and analyte gas molecules. Illustrations demonstrate current conduction under **a** forward bias and **b** reverse bias. The difference in sensing can be observed as there is considerable device current under forward bias in comparison to that of reverse bias
(vi) In reverse bias, the diode current is very low, i.e., diode saturation current. This current is low because of the large depletion region exists at the junction. Now, in presence of VOCs, though electrons will be released by the oxygen adsorbates, but they will fail to cross the junction. Thus, no appreciable response was found in reverse bias case. This is explained schematically in Fig. 8b.

## Conclusions

Thus, in this work, it was established that it is possible to fabricate a single NiO/ZnO-based vertical heterojunction diode via chemical based, cost-efficient hydrothermal technique that can selectively sense different VOCs by tuning the forward bias voltage (in the nonlinear region). Selective sensing of 2-propanol (19.1 times for 95 ppm with response and recovery times of 70 s and 55 s, respectively, at 3 volts), toluene (20.1 times for 95 ppm with response and recovery times of 100 s and 60 s, respectively, at 4 volts), and formaldehyde (11.2 times for 95 ppm with response and recovery times of 88 s and 54 s, respectively, at 5 volts) was achieved using this device. The sensing mechanism demonstrated that the exponential rise in carrier concentration with increase in bias voltage (resulting in increasing surface carriers) can be related to the type of gas being sensed by the device, as it depends on the electron requirement of the analyte gases. We believe, diode sensor can play an important role to develop selective, highly response VOC sensor in near future thereby eradicating complex structured gas sensor arrays being used commercially nowadays.

## Supplementary information

Supplemental information
