# Supplementary material for: Voltage-controlled NiO/ZnO p–n heterojunction diode: a new approach towards selective VOC sensing
Source: Microsyst Nanoeng. 2020 Jun 1;6:35. doi: 10.1038/s41378-020-0139-1 (PMC8433462; doi:10.1038/s41378-020-0139-1)
Supplement: Supplementary file 1 — Supplemental information [file 41378_2020_139_MOESM1_ESM.pdf]

Voltage controlled NiO/ZnO heterojunction device: A new approach towards selective VOC sensing

Sayan Dey[1], Swati Nag[2], Sumita Santra[3], Samit Kumar Ray[3, 4*], Prasanta Kumar Guha[1, *]

<u>Electronic Supplementary Information</u>

Relation between top layer carrier concentration and device current

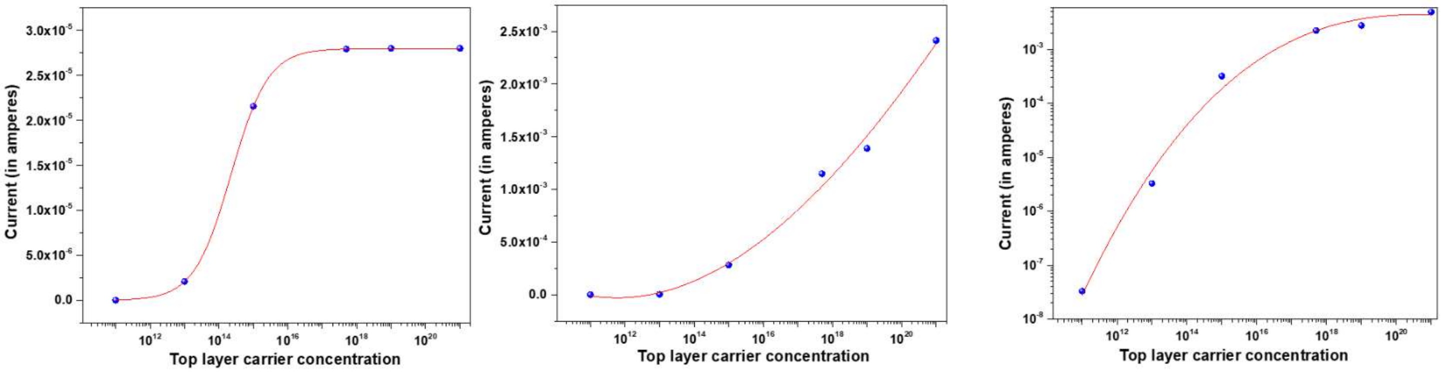

(a) (b) (c)

Fig 1S: Relation between top layer carrier concentration and device current when operated under forward bias at V_{bias} of (a) 3 volts (b) 4 volts and (c) 5 volts

For a gas sensor device, the top layer carrier concentration determines the rate of current flow. The current flow for NiO/ZnO device was therefore expected to vary differently under different bias owing to the non-linear nature of the device. From the above figure, the difference in nature of the three plots clearly proves the non-linearity of the device aiding to voltage controlled selectivity.

Comparison between simulation and experimental results

The I-V characteristics obtained from device simulation (in Silvaco) and during experiment were compared below. It was observed that the simulated and experimental plots show very less deviation thereby proving the authenticity of the predicted models.

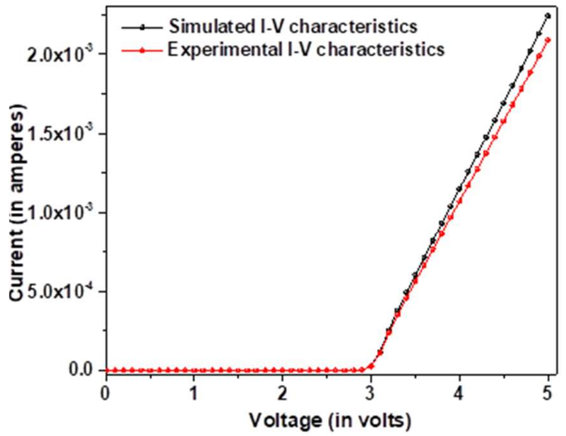

Fig 2S: Comparison between I-V characteristics of the NiO/ZnO heterojunction device: simulated (in black) and experimental (in red)

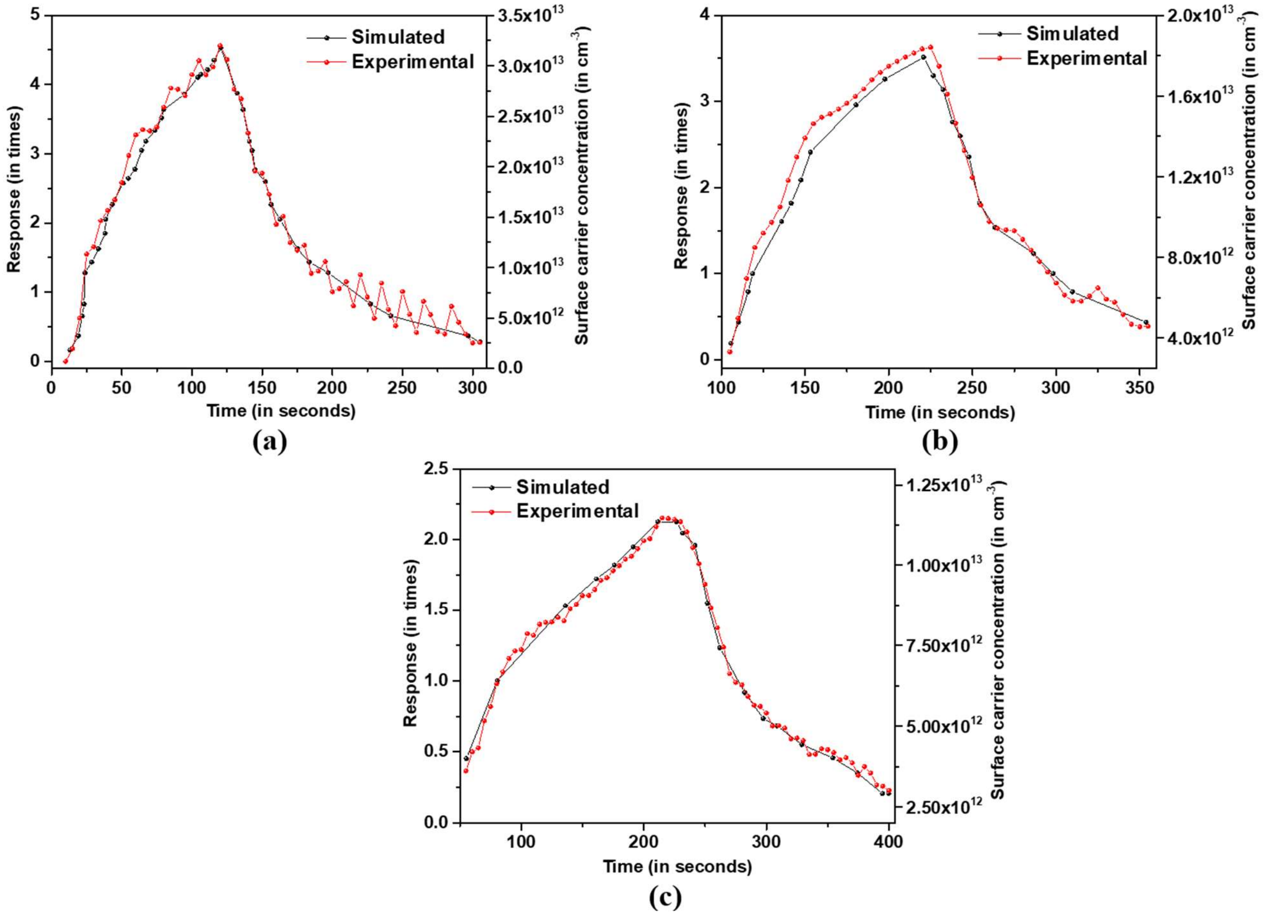

Fig 3S: Comparison between transient characteristics of the NiO/ZnO heterojunction device: simulated (in black) and experimental (in red) for 19 ppm of (a) 2-propanol in 3 volts (b) toluene in 4 volts and (c) formaldehyde in 5 volts

The fig 3 shows one transient cycle (at 19 ppm of VOC) for each of the three VOCs in their respective operating voltages. It is observed that the simulation result is in good agreement to the experimental results.

Selectivity of sensor in cut-off region

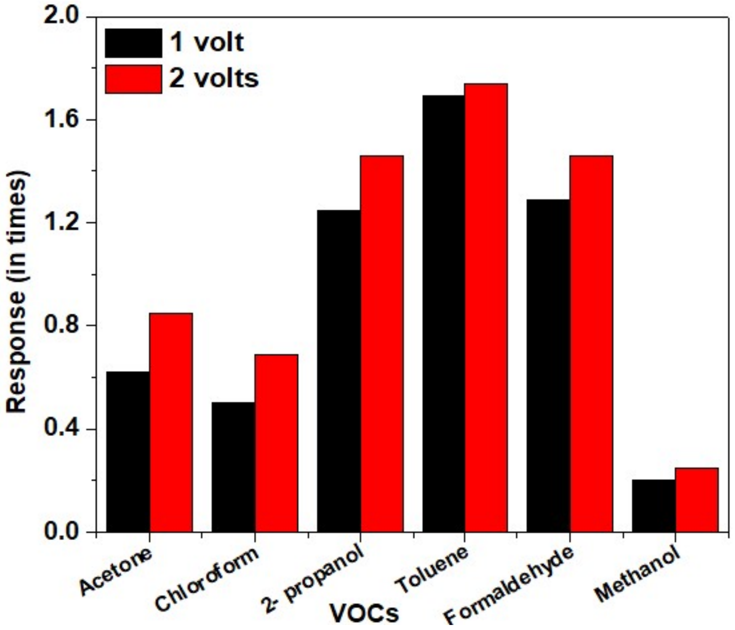

Fig 4S: Selectivity of the sensor device in the cut-off region (i.e. 1 and 2 volts)
